# Descriptive and Illustrated Catalogue of the Physiological Series of Comparative Anatomy, Contained in the Museum of the Royal College of Surgeons in London

**Published:** 1843-01

**Authors:** 


### ART. XVIII.

*Descriptive and Illustrated Catalogue of the Physiological Series of Comparative Anatomy, contained in the Museum of the Royal College of Surgeons in London.—London,* 1833-41. Five Vols. 4to, pp. 1244. With numerous Plates.

SOME of our readers may perhaps think a catalogue an inappropriate subject for review; but when it is considered of *whose* collection this catalogue gives an account, and by *whose* labour it has been perfected, we are sure that even they will feel our notice of it to be called for, not merely as directing their attention to the treasure they possess, nor simply as pointing to the amount of information to be derived from this description of it, but as an appropriate tribute of gratitude for the patient devotion, on the part of Mr. Owen, of his eminent talents, during so many of the best years of his life, to the completion of this object for the benefit of the public.

But the object was not unworthy of him. It is known to almost every one that the plan of the museum was formed by John Hunter; and that by far the larger number of specimens were collected and prepared by himself. We have noticed in a former volume (vol. IV. pp. 83-6) the untiring zeal and the disregard of all considerations of self-interest, which carried him forwards in the prosecution of his vast work, that of building up the science of physiology on the sure foundation of comparative anatomy. This he regarded as the great object of his life; and for this he will ever be entitled to the tribute of admiring gratitude from all who profit by his labours; and whom will not this expression include? It has been too much the fashion, even in this country, to refer to the school of Cuvier as that in which the important truth was first broadly enunciated, that no functional operation of any living being can be completely understood, until it has been compared with the corresponding operations of all others. And this belief has been prevalent, not only among the large class who are satisfied with a general notion of the matter, but among some who have professed to be really well acquainted with the grounds of it. True it is, that in successive Hunterian orations attempts have been made to correct this misconception; but the statements therein contained have been looked upon with a not unreasonable suspicion, as panegyrics dictated by time, place, and circumstance, and uttered by admiring and grateful pupils. The work before us, however, will afford convincing evidence to all who merely glance cursorily through its contents, that we are right in claiming for Hunter the foremost place amongst the modern *illuminati* of physiological science; and foremost, not merely in point of time, but in the grandeur of his conceptions, and the scope of his labours. For, whilst the labours of the Cuvierian school have been principally restricted to the study of animal structures, it is evident that Hunter's mind already conceived the idea, which has recently proved so fertile in important results, that the study of the structure and vital actions of plants is no less important as a foundation for those generalizations, which alone can raise physiology from the rank of a crude unarranged collection of imperfectly-understood facts, to the level of a stable and well-ordered science. In proof of our assertion, we can now appeal to the Hunterian Museum, a monument to the fame of its great founder, which will outlive, we venture to predict, those works on which the fame of Cuvier is founded. For whilst the progress of knowledge is gradually impairing the value of the "Règne Animal" as a key to classification, and whilst new methods of research are shortening the laborious path by which the great principles of the "ossemens fossiles" may be applied, every addition to the Hunterian Museum tends but to render it a more perfect representation of the design of its founder, and to afford new materials for those laws of animated nature which it was his aim to discover. It is not our present purpose to institute a formal comparison between the minds of these two illustrious men; but we may briefly express our opinion of their respective characters, in saying that, whilst to Cuvier we would assign the highest *talent*, the brilliancy of that intuitive *genius* which makes the great poet, the great artist, or the original discoverer, is chiefly to be found in Hunter. The discoveries of Cuvier were made in the true spirit of inductive philosophy, by the collection of instances and the generalization of particulars. The *idea* of applying this philosophy to the science of life, by extending the primary sources of inquiry, from the few animals of not very dissimilar character to which appeal had been previously made, to the whole range of the animal and vegetable kingdoms, is unquestionably Hunter's; and we have good reason to know that Cuvier profited largely by it.

The Hunterian Museum was purchased by a Parliamentary grant of £15,000, and was committed to the charge of the College of Surgeons, on their undertaking its conservation, and also agreeing to prepare a catalogue of its contents, and to give every facility for the beneficial employment of them by the public. Further grants, amounting to £27,500, were afterwards made for the erection of a building for its reception; but the recent extension and reconstruction of this building has been accomplished by the funds of the College only, and reflects the highest credit upon those under whose direction it has been effected.

That the College has nobly performed its promise of "preserving the collection in the best possible state at their own expense," cannot be questioned. Not only has there been the most sedulous care to preserve, with an almost religious veneration, every preparation which was left by Hunter, but a very large number of important additions have been made to the collection, not only by the donations of the scientific public, but by the labours of the conservators, especially Mr. Clift and Mr. Owen. In a large proportion of instances, these additions have been of the most important and interesting character; some being designed to fill up lacunæ which Hunter had himself indicated, but which he had not the opportunity of completing; and others being derived from various remarkable animals which have been discovered since his death, such as the ornithoryncus, echidna, dugong, squalus maximus, &c.

"The [physiological series of the Hunterian] collection includes 3745 specimens of that kind which require the utmost skill and science in their preparation, and the greatest care and expense in their preservation. That Mr. Hunter, however, regarded it but as an approximation to an adequate display of the general plan which pervades organic nature, is to be inferred, from the earnest assiduity with which, to the last day of his existence, he laboured towards its perfection. Some deficiencies he has himself noted, and occasionally has indicated the animal in which would be found the intermediate gradation of structure necessary to complete a series. The additions, therefore, have been prepared in exact accordance with those indications, and always, it is hoped, in harmony with the founder's original design. As it is important to the history of physiology, and just to the memory of Mr. Hunter, to maintain the integrity of his collection, these additions are so marked, that they are at once readily distinguished from the Hunterian specimens, and the original condition and connexion of the latter left undisturbed." (Preface to vol. i. p. xi.)

By a separate index to these additions, which is appended to the last volume of the catalogue, we are enabled to estimate their number at about 706, exclusively of Mr. Swan's munificent donation of his series of preparations of the nervous system, which was not received until after the publication of that portion of the catalogue which embraces the nervous system. Of these additional preparations, 186 have been made by Mr. Clift, 220 by Mr. Owen, and 66 by Sir Everard Home. The subjects of forty-eight of them were liberally presented by the council of the Zoological Society, which has shown the greatest readiness to contribute to the perfecting of this splendid collection, at the expense of its own museum.

The utility of this collection to the public, however, has until recently been much less than the public had a right to expect, after having aided the College with grants amounting to £42,500, for the purpose of having it preserved in the most advantageous manner, and of having the necessary facilities afforded to those who desired to study it. No printed catalogue was issued; and the visitor has been obliged to depend upon the information he might be fortunate enough to obtain from the conservators and their assistants, upon whose time and attention the demands were too numerous for any individual to expect much assistance. We cannot but deem the conduct of the council very blameworthy in this respect; but the neglect of the past has been amply redeemed by the recent completion of a catalogue which reflects the highest credit on all concerned in it, and which supplies all that could be desired or expected, whether to the student who seeks to become acquainted on the spot with the individual preparations, or to the physiologist who wishes either to take a general survey of the contents of the museum, or to be directed to the study of any particular department. We can scarcely speak more highly of this great work, than by saying that it is such a catalogue as Hunter himself could scarcely have surpassed, had he not been prevented from thus completing his grand design by his sudden death. The vast amount of knowledge requisite to construct such a catalogue, and the immense labour which must have been bestowed upon it, will be best understood, when we have given a sketch of the history of the work, founded upon the materials contained in the prefaces to the first and last volumes.

Before the formation of the present catalogue, the printed works from which information could be derived respecting the physiological collection, consisted of the published writings of Hunter, a brief synopsis of the contents of the Museum published in 1818, and the lectures on comparative anatomy of Sir E. Home. The latter profess to explain the Hunterian collection; but they contain descriptions of a small number only of the preparations, and these descriptions are unaccompanied by any reference to the particular specimens. The synopsis is a mere table of contents, which was in many instances arranged upon false principles, and exhibited but a very imperfect knowledge of the true relations of different organs to each other. This synopsis has consequently been widely departed from in the present arrangement; the alterations, however, consist for the most part of a return to Hunter's original arrangement, and have been either suggested by the Hunterian manuscript catalogues, or made with the view of obtaining greater simplicity and consistency, and a more regular subordination in the several groups of preparations.

The catalogues descriptive of the original preparations have hitherto existed only in manuscript. The very imperfect materials which they have afforded for the identification of the specimens will be obvious from the following enumeration of them:—1. A manuscript catalogue in Hunter's handwriting, without date, but probably written soon after his return from Portugal in 1763. It briefly defines the nature of about 200 specimens; of which part were of a physiological and pathological character, and part zoological; the latter having been for the most part collected in Portugal, Spain, and Belleisle, during his service as an army-surgeon. This collection was the germ of the future Hunterian Museum, and the foundation of its several departments.—2. A small octavo manuscript without date, in the handwriting of Mr. W. Bell and of others who assisted Hunter; this describes or notices 561 of the physiological preparations.—3. The quarto catalogue, which is the most valuable of the original Hunterian documents relating to the present department of the collection. It consists of twenty thin fasciculi, respectively devoted to the several classes of organs described; and it is in the hand-writing of Mr. W. Bell and others, with additions and corrections written by Hunter himself. Most of the fasciculi commences with general observations on the series of organs to which they respectively relate; and these valuable expositions have been introduced, with a few verbal corrections merely, in their appropriate places in the present catalogue. Still the chief importance of this catalogue consists in the information which it supplies respecting the scheme of arrangement, and the general physiological principles intended to be illustrated by the different series. The descriptions of the individual preparations are comparatively few; and these, for the most part, are confined to a brief definition of the object. Many had merely the name of the animal or part written on the top of the bottle; and the rest were without either name or number.—4. From these materials, Dr. Baillie, Sir Everard (then Mr.) Home, and Mr. Clift commenced, in the year 1793, the formation of the folio catalogue, which served for the use of visitors until the publication of the present one.—5. The collection having been rearranged and renumbered by Mr. Clift, under the direction of Sir E. Home, in 1817, a new catalogue was commenced by the former gentleman, which included, with the original memoranda attached to the specimens, some notes of his own as to their history. This document has proved of material use in the determination of many of the unnamed specimens; but it is only of very limited extent.—6. Another document of much importance in the identification of the individual specimens, is a catalogue by Hunter of a series of drawings chiefly taken from preparations in the museum, and intended to illustrate their description. About thirty specimens, in some instances of complicated and minute structures, have been determined by this mode of comparison.

In the present catalogue, the original Hunterian descriptions have been retained, as far as possible ; additions have been made to them when they were found to be not sufficiently clear; and new descriptions have been given of all the remaining preparations. The information most commonly required in addition to the previous descriptions and notices, has been the name of the species of plant or animal from which the preparation had been derived. Where this information is attempted in the manuscript catalogues, it is commonly of a very imperfect nature, such as "a monkey," "a whale," "a beetle," or "a snail;" or the indication is still more vague, as "an insect," a "sea-worm," "a shell-fish," &c. In a great proportion of the specimens, the description relates only to the organ, or ends with "animal unknown." Hunter cannot be justly blamed for the want of this kind of information; since many of the animals he dissected were unknown to naturalists at that period, and consequently were without cognizable or scientific denominations. In many cases, where the species is more definitely indicated, rectification of the name has been found necessary; and this has been effected in some instances by a comparison of the preparation with such descriptions and figures of the anatomy of the animal from which it was supposed to be taken, as could be found in print; and in other instances by actual dissection of the animal. The former mode of identification, even in the more entire specimens of dissected animals, is necessarily very limited; since in general the preparations are more or less mutilated in parts of the body from which the zoological characters are derived, so that a satisfactory identification from books is sometimes entirely precluded. The assistance derivable from the same source, in ascertaining the species to which the unnamed specimens of detached organs belong, is still more casual and uncertain ; so that in their elucidation it was necessary to consult the book of nature. More than two hundred dissections have been made with this view, especially of such animals as, from any indication in the manuscripts, appeared to have afforded a doubtful specimen; and many which were before unknown have been in this way identified. At first, the means of instituting these comparisons were few, and depended on the casual acquisition of animals from uncertain sources. But since the institution of the Zoological Society of London, a more ample scope for the investigation of comparative anatomy has been opened; and the materials for these progressive inquiries have been afforded with the greatest liberality.

From these statements some idea may be formed of the skill and labour required to bring this great work to its present high state of perfection. The Council have most laudably determined that it shall be brought before the the public in a manner every way worthy of its great authors, John Hunter and Richard Owen; and the volumes are not only distinguished for the typographical elegance and accuracy which characterize the works that issue from the press of their learned printer, but are adorned with a series of most beautiful engravings of some of the most perfect anatomical drawings which it has ever befallen us to see. With the exception of two, from the pencil of Mr. Owen, illustrative of the anatomy of the nautilus, and six illustrating the comparative anatomy of the organ of hearing, which were presented to the College by Sir A. Carlisle, the whole series, seventy-eight in number, are engraved from the original drawings of William Bell and J. Van Rymsdyk, two artists whom Hunter kept in his service for several years. The drawings of the former are remarkable alike for their artistic merit, and for their anatomical precision. It is evident that he well understood the structures he was delineating, and looked upon them with the eye of an anatomist, as well as that of a draughtsman. We cannot conceive anything more beautiful than his representation of the anatomy of Holothuria, (plate iii. vol. i,) to which ample justice has been done by the engraver, Mr. Basire; or than the series illustrating the development of the egg, which is contained in the last volume. Of many others, by the same artist, the subjects afford less scope for the display of talent; but all are treated with like skill. The drawings of Van Rymsdyk are perhaps superior in force and boldness, but appear to us less perfectly finished than those of his coadjutor. We shall presently return to the subjects of some of these delineations, which present topics of peculiar interest.

We must not conclude our general notice of this work, without adverting to the elaborate double index at its close. There is first an anatomical index, in which the subjects are classed in the same manner as in the body of the work, i. e. according to their physiological character; this is, therefore, rather a very detailed table of contents. Under every subdivision are given references to the numbers of the preparations which illustrate it, and to the volume and page of the catalogue in which these are described. By this the physiologist will be able to ascertain at a glance the materials which he will find in this museum for the elucidation of any particular department of his inquiries. The other index is constructed upon a zoological arrangement; so that by a reference to the name of any species (which will be easily found by turning to its class and order) the comparative anatomist is informed of all the preparations which illustrate the details of its structure. The preparation of this index must have been a work of immense labour; but we feel sure that its compiler will be amply repaid by the increased facility which is thus afforded in the consultation both of the preparations themselves, and of this descriptive Catalogue of them. This index further affords some interesting information respecting the large number of species from which the materials of this noble collection have been derived. As might be expected, a considerable proportion of the preparations have been made from the bodies of the larger and more accessible animals; but, in each of the higher groups, we find that one species at least has been submitted to detailed analysis; so that a very complete series of the chief types of structure is presented to us for every organ. Of course man stands at the head, in point of the number of preparations derived from his body, as well as in zoological arrangement. Several preparations of the chimpanzee and orang-utan, which were rare animals in Hunter's time, have been added by Mr. Owen. The hedge-hog and the mole, among the Insectivora,—the lion, cat, dog, and seal, among the Carnivora,—the porpoise, dolphin, bottle-head, piked and whalebone whales among Cetacea,—the elephant, horse, and pig, among the Pachydermata,—the camel, fallow-deer, sheep, and ox, among Ruminantia,—the rat, beaver, porcupine, and rabbit, among Rodentia,—the opossum and kangaroo among the Marsupialia,—furnished Hunter with a large number of his specimens. To these we may add the ornithorhyncus, an animal unknown to Hunter, whose extraordinary structure has been displayed in a series of seventeen preparations made by Messrs. Clift and Owen. Yet the total number of species among the Mammalia alone, exceeds 150. In the class of Birds, again, the owl, sparrow, cuckoo, fowl, ostrich and goose, furnish a large quota; but the total number of species that have furnished preparations is 70. Among Reptiles, we find the turtle, crocodile, guana, chameleon, rattle-snake, boa, frog, and siren, particularly illustrated; whilst the total number of species included exceeds 70. Among Fishes, the species whose structure is most completely displayed by Hunter, are the gray shark, dog-fish, skate, electric ray, sturgeon, mackerel, cod, conger eel, electric eel, and lamprey; to these may be added the great basking shark, of which numerous interesting preparations have been made by Mr. Clift. The total number of species of fishes, of which preparations are included in the museum is nearly 80. It will be evident to any one in the least conversant with zoological arrangement, that better types of the several groups could scarcely have been selected, than those which circumstances threw in Hunter's way. But so far from being satisfied with these, he used every exertion and spared no expense, to obtain specimens of the rarest and most curious animals; and the value of the collection to the naturalist, as well as to the comparative anatomist and physiologist, is thus immeasurably increased.

Until we had consulted this zoological index, we had ourselves no idea of the extent of Hunter's labours in the anatomy of the invertebrata. It must be borne in mind that, at the period when he was engaged in prosecuting them, there was little or nothing known with respect to the principles of their classification, and this was a consequence of the absolute deficiency of any satisfactory information as to the leading types of structure which prevail among the residents in this vast province of the animal kingdom. Thus, in the system of Linnæus, which was the only one at that time in general use, all the invertebrata which present an evidently articulated structure, such as true insects, crustacea, arachnida, and myriapoda, are grouped together as insecta, whilst all in which this structure is not obvious, are associated together as vermes. It is impossible to imagine anything more heterogeneous than this last group, for it brought together in close proximity many really vermiform animals that belong to the articulated series, the whole sub-kingdom of the mollusca, all the radiata, and even some of the lowest fishes in which the vertebrated structure is indistinct. To Cuvier is usually assigned, and not unjustly, the merit of first breaking up this group into its proper divisions; and of showing that the articulated, molluscous, and radiated types of structure are equal in rank to the vertebrated; so that the animal kingdom must be primarily divided into these four sub-kingdoms. Yet we think that the evidence we possess in the museum and fragmentary works of Hunter, is quite sufficient to show that he perceived the necessity of an entirely new system, that he was diligently collecting materials from which to construct it, and that so extensive and accurate were his researches, he could scarcely have failed to strike upon the true principles, had his life been prolonged sufficiently to give to the world the generalized results of his wonderful labours. Of this there is evidence in his arrangement of his preparations of the "nervous system;" in which he distinctly points out those entirely different types of structure, characteristic of the mollusca and articulata respectively, of which so important a use is made in the "Règne animal" of Cuvier. Indeed we are in possession of evidence from a private source, that the Hunterian Museum was made the object of particular study by Cuvier, previously to the publication of the first edition of the "Règne animal." Yet with that jealousy that too often shows itself among our Gallic neighbours, Cuvier never spoke of Hunter but as a second or third rate anatomist, classing him with or even below Camper and Vicq D'Azyr; and referred to him only as the author of a treatise on the teeth, and some papers in the Philosophical Transactions. Yet that Hunter had long anticipated Cuvier in many of his most interesting observations upon invertebrated animals, the preparations and drawings he has left afford ample evidence. Thus the anatomy of several of the tunicated mollusca had been investigated by him in a way which leaves but little to desire; he had thoroughly examined the solen, sepia, and many gasteropods; and has left the most elaborate dissections and figures of the cirrhipeds; in all which he was but followed by Cuvier in those "Mémoires sur les Mollusques," on which his reputation as an original inquirer greatly depends. The figure which Hunter has left of the circulation in the chlœia capillata, a red-blooded worm, far surpasses in beauty and detail any of those with which Cuvier illustrates the memoir dedicated to what he regarded with his latest breath as one of his most interesting discoveries. The illustrations of the anatomy of the echinodermata have not, until the recent monograph of Valentin upon the echinus, been surpassed either as to minuteness or accuracy; and, excepting in the disputed article of the nervous system, little is added in the elaborate and well-known monograph of Tiedemann, to the anatomy of the Holothuria, as it is displayed by Hunter. The total number of species of invertebrated animals of which the museum now contains preparations exceeds 300; and although part of these have been more recently added, the number of species dissected by Hunter himself could not have been far short of 200. We have no hesitation in saying that this number exceeds that which could have been furnished by the united labours of all the other anatomists of that or preceding periods.

We shall now present our readers with a cursory sketch of the arrangement of the Museum, and with a few selections from the Descriptive Catalogue, which will afford a fair sample of the plan of the work, and of the information that may be derived from it, even by those whose opportunities of consulting the great original are few and far between.

The series of Preparations is disposed in two divisions; first, those illustrative of the functions which minister to the necessities of the individual; and secondly, those which provide for the continuance of the species. The First Division commences with a few examples of the component structures of organic bodies; and then extends into a series embracing the active and passive apparatus for progressive motion. This series begins with specimens of the chief examples of Motion in Vegetables—the mimosa, dionœa, hedysarum, and erythrina; and in regard to the analogy between these and muscular movements of animals, it is evident that Hunter possessed ideas not far from the truth. The structure of Muscle is the next subject embraced in this series; and among the preparations illustrative of it is one whose curious history is well known to professed physiologists, but which we shall quote as it may be new to many of our readers:

"34. The biceps flexor cubiti muscles from the arms of a Negro. That of the right side is in its natural state; the other shortened one-half of its length, in consequence of the os humeri of that arm having been fractured obliquely, and having become united with the fractured ends of the bone riding on one another. After the union of the bone, the biceps (together with the other muscles of the arm) became shortened by the interstitial absorption, so as to correspond with the diminished length of the bone, and the arm regained its natural action." (Vol. i. p. 9.)

The history of the case may be found at greater length in Sir E. Home's Lectures on Comparative Anatomy; in which it is mentioned that, on the man's death occurring some years after the accident, the biceps muscles of both arms were carefully dissected out, and being measured, the one was found to be eleven inches long, the other only five, so that the muscle of the fractured arm had lost six inches. Scarcely a more beautiful instance could be adduced of that tendency to the advantageous reparation of injuries with which the animal body is endowed,—a tendency conveniently referred by some to the exertions of an ill-defined semi-intelligent principle, *vis medicatrix naturæ*, but in reality only one result of those laws of nutrition by which the fabric is continually preserved in spite of the waste it is as constantly undergoing.

After the series illustrative of muscular structure and action, we find another exhibiting the application of Elastic Structures—either as antagonists to muscles, as in bivalve shells, or as aiding muscular action, as in the ligaments of the spinal column of vertebrata. Following this is a short series exhibiting the different substances of which the Skeleton is composed; and then commences the series of hard structures which serve as passive instruments of locomotion. Of the first part of this, embracing the calcareous fabrics of zoophytes, mollusca, and crustacea, under the general term Shell, a comparatively small number of the preparations were the work of Hunter himself; many of them were presented by Mr. Hatchett, whose memoir on the subject in the Philosophical Transactions of 1799, (from which extracts illustrative of the preparations are included in the Catalogue,) is still of standard authority; and many have been more recently added. The next division illustrates the structure and growth of Bone; and among these we find the two following experimental results, which are peculiarly worthy of notice:

"188. The left tarsus of the domestic fowl, upon which the following experiment was performed. Two small holes where made by cauterization near the extremities of the bone; the length of the bone at that time being two inches aud ten lines, and the distance between the holes one inch and eight lines. After a certain period the animal was killed, and the length of the bone was found to be three inches seven lines, while the space between the apertures was one inch and eleven lines; the increase of the bone beyond the points of cauterization being more than double that of the space included between them.

"189. The right tarsus of the domestic fowl, longitudinally bisected, to show the results of the following experiment. When the animal was young, the bone was perforated near each extremity, and a small leaden shot was introduced into each hole. After a certain period the animal was killed, and the length of the bone was found to have increased to three inches and ten lines; but the distance between the shots which had now reached the medullary cavity was exactly the same as when first introduced." (Vol. i. p. 41.)

Upon these experiments Hunter founded the statement contained in his Lectures on Surgery, that "a bone does not grow in all its parts; that is, it does not grow by addition of new particles among those already arranged, or in their interstices, but by the addition of parts lengthways or sideways of the bone;" a statement which, if not absolutely true, is certainly not very far wrong. Late researches upon the structures of the hard parts of the invertebrata have shown that they too are organized, and that they differ chiefly from bone in the absence or imperfect possession of that vascular network which in the latter lines the Haversian canals, but does not pass into the actual substance of the bone. Many other interesting preparations, which we cannot stop to notice, are contained in this series.

The next principal series is a very interesting one illustrative of the composition of the skeleton, with reference to its articulations, and to the admixture of osseous, cartilaginous, and ligamentous structures. In this are contained some of those preparations on which were founded Dr. William Hunter's accurate descriptions of the structure of articulate cartilage; he was, we believe, the first to notice the continuity of the synovial capsule over the surface of the cartilage, thus demonstrating it to be a shut sac like the serous membranes; and he also noticed, under the name of *Circulus articuli vasculosus*, the peculiar anastomotic disposition of vessels around the margin of the joint, which has been recently described by Mr. Toynbee. Following this series is one illustrating the "mechanical contrivances by which the powers of the muscles are augmented;" and another comprehending "the various organs for progressive motion." In the latter are some interesting preparations, added by Mr. Owen, which illustrate the mechanism of the movements of serpents, which use the free extremities of their ribs in crawling along the ground, very much in the same manner as the centipede uses its legs. The proper muscles of the spine, being so largely concerned in the movements of these animals, attain a very high degree of development. Distinct analogies may be recognized between their several groups, and the spinales and semispinales dorsi, the longissimus dorsi, the sacrolumbalis, the multifidus spinæ, and the interspinales and intertransversales of man. External to the multifidus spinæ is a series of short and strong oblique muscles, which, like the levatores costarum, arise from the transverse processes, and are respectively inserted into the rib attached to the succeeding vertebra. Where these are inserted, longer muscles, *prætrahentes costarum*, arise, which run more obliquely backwards, each of which terminates in the eighth rib beyond that from which it arose, but is also inserted into all the intermediate ribs, and is closely connected with the intercostales. The muscles on the inferior surface of the spine are peculiarly developed; they arise from the transverse processes, and converge forwards to be inserted into the inferior spinous processes of the vertebræ. External to these are situated the *retrahentes costarum*, which arise from the lower part of the transverse processes; and after passing obliquely forward over three ribs, are respectively inserted into the fourth. Beneath these, there is a stratum of short muscles, which arise respectively from the head of one rib, and run obliquely backwards to be inserted into the next rib; and there is another series, analogous to those which Winslow has termed sub-costales in the human subject, which form a kind of continuation of the retrahentes, arising where they terminate, and passing on to be inserted into the second rib beyond. The *retrahentes* and their assistants are manifestly intended to advance the spinal column by drawing back the ribs when their points are resting on the ground, whilst the *prætrahentes* bring the ribs again forwards when the body is at rest.

The second subdivision embraces the Organs of Digestion; and at the head of these are placed the preparations illustrating the structure and growth of Teeth. This position, which we regard as peculiarly appropriate to them in a physiological arrangement, was assigned to them by Mr. Clift. In the time of Hunter, when the teeth were usually enumerated by anatomists among the bones of the skeleton, probably no other physiologist would have thought of classifying them, as he did, with hairs and horns. Some of their striking relations to the extra-vascular productions, thus early appreciated by him, have been subsequently insisted on by other philosophical anatomists, to the exclusion of the facts and arguments which are still valid for regarding them as appendages to the osseous system. Sir E. Home removed the series in 1818, to a place between the "stomachs" and "intestines," a most absurd position. The present locality is peculiarly appropriate, as in harmony with the structural relation of teeth to bone, and with their physiological action in the economy. The series commences with a very interesting collection of parts analogous to teeth, including the various structures answering the same purposes in invertebrate animals, together with the bills of birds, and whalebone: of this last substance there are several interesting preparations; and illustrative extracts are introduced into the Catalogue from Hunter's paper on the structure and economy of whales, in the Philosophical Transactions. The series of teeth proper is very extensive, illustrating their formation, component parts, shedding, and situation. At the commencement of the series illustrating the structure of the Stomach are some very interesting original observations of Hunter's, which show that he had long anticipated in idea the celebrated French definition "*Un animal est un estomac servi par des organes.*"

"The apparatus necessary for the simple operation of digestion is as simple as anything we can well conceive. It only requires a bag or cavity fit to contain the substance to be digested, joined with the power of furnishing the fluid capable of digesting or animalizing the said substance. In such a light it is only to be considered as a gland with a cavity. But it was necessary that there should be some part added to furnish this bag with materials to be digested; for which purpose there are in some arms, in others both arms and teeth, &c. Besides the simplicity of the apparatus for the operation of digesting, there is another apparatus added to fulfil the intention, which is the system for absorbing the animalized parts for the nourishment of the same bag; and added to this power of secretion and absorption is the power of throwing out of the bag the indigestible parts, acting as a kind of secretory duct. Nothing more is necessary to complete an animal than the power of continuing the species, which power is superadded to this bag in many. From this account nothing can be more simple; however, it completes a whole animal; and nothing more can be necessary for the support of such an animal; but when we come to such stomachs as have parts superadded for other purposes than the above, then we find that this same apparatus for digestion has also parts superadded for the purpose of digesting; so that the parts preparatory and subservient to digestion become more complicated, and indeed so much so that there is hardly any system in an animal more complicated in itself; and when we consider the varieties of these complications which take place in the various animals, they appear to be almost without end. It is these complications and varieties that we mean to consider, and reduce, as far as they will admit, to their several classes." (Vol. i. pp. 112-3.)

We do not admit this statement in its full force; since we think it would be easy to prove that the essential characteristic of an animal is its possession of consciousness and voluntary power; the instrument of which faculties is a nervous system; and, that the presence of a stomach and its appendages is necessarily connected with the peculiar conditions of animal existence, such as the power of locomotion, and the nature and sources of the food. But the beauty and truth of Hunter's views, considered with reference to the digestive system only, must be apparent to all; and of their novelty at that time we do not think there can be the least doubt. No previous anatomist, we feel sure, had succeeded in bringing together so extensive a series of preparations illustrating the various forms of the digestive apparatus, from the simple sac of the hydatid or polype, to the complex intestinal canal of the higher vertebrata; and no one, therefore, could have possessed such clear views of the true relations and analogies between parts apparently different, as those at which Hunter had arrived. We cannot stop to notice any of the very numerous interesting preparations contained in this series, except No. 523. "The stomach of a sea-gull, which had been brought to feed on barley, showing that the muscular parietes of the gizzard were become much thicker in consequence." The history of this preparation is given in Sir E. Home's Lectures; and from it we learn that the bird had been kept by Hunter for a year. It is to be remembered, however, that the gull and many birds of its order are naturally to a certain extent omnivorous, being destined to act as the scavengers of the sea-shore, as well as to feed on living fish; and that an alteration in its natural habits might be allowed by the adaptive power of its structure, which might not be as well borne by more purely carnivorous birds. We learn from Hunter's observations on digestion, that he gradually brought a kite to feed on bread alone; but the affinity of this article to flesh is too close, both in respect to its composition and consistence, for the experiment to be a satisfactory one; and we doubt if any raptorial bird could be brought to feed on grain alone.

Upwards of 450 preparations are contained in the series of digestive organs, exclusively of those of the teeth; and the account of these occupies the whole remainder of the first volume, except the part devoted to the descriptions of the plates. Of these, the first two include figures taken from preparations of bones of the hog, in a growing state, coloured with madder, and forming part of the series preserved in the collection to illustrate Hunter's theory of the growth of bone. They are very interesting as memorials of the original appearance of these preparations, the colours of which have now faded. Plate iii. is from the beautiful drawing of the holothuria to which we have already alluded; and it is accompanied by the full original description which has been fortunately preserved to us. The following introductory observations show how much of the philosophy of comparative anatomy was in Hunter's mind, although often obscurely expressed:

"Perhaps there is nothing so difficult in natural history, as the finding out the uses of the different parts of animals when they differ widely from those we are best acquainted with, especially so in those whose economy we can hardly observe. If the regular gradations from one species or genus of animal into that of another was well ascertained, we could most probably assign the uses to each part from analogy; but as that gradation is not yet known, and as we often are examining animals that seem in many of their parts to have no affinity to any animal we know, for what answers any one purpose in the animal economy and may exist in most animals, yet shall so vary in its form as not to give the least idea of its use,—therefore we are left to conjecture about their uses in such animals. The priapism is an instance of the above observation; many parts can be made out; but the uses of many must be left to conjecture till the analogy is made out complete." (Vol. i. p. 251.)

In this as in many other cases, it is evident that Hunter was well aware of the essential character of a gland, as since described by Müller; for he correctly refers to the "appendicula cæca which surround the mouth and fauces and enter their cavity," as "salivary glands and ducts."

In the succeeding plates are almost equally beautiful delineations of the structure of the Barnacle, (which has been since described in the splendid work of Poli, who attended Hunter's lectures and enjoyed his friendship whilst resident in London,—as well as by Cuvier,) and also of the Ascidian Molluscs, the true nature and relations of which were well known to Hunter, who proposed to distinguish them as a distinct group under the name of "soft-shelled," which more truly accords with their real character than "shell-less," as they have been subsequently designated by Cuvier. Afterwards there follow five plates representing the digestive apparatus of birds, and that of the crocodile; which last is observed by Hunter to bear a strong resemblance to that of birds in the disposition of the central tendon, and of the turns of the duodenum.

The second volume contains the Absorbent, Circulating, Respiratory, and Urinary Systems. The description of the preparations illustrating the first is prefaced by a full statement, in Hunter's own words, of his views respecting absorption; as these, however, are sufficiently well known through other channels, we need not stop to quote them. It is right that we should remark, however, that Hunter's doctrine—stated by himself in the following terms, "The arteries perform the different and immediate actions of the machine as before; the veins replace the blood for the repetition or continuance of these actions as before, but have now lost their power of absorbing; the absorbents exclusively perform those actions, a summary of which we have previously given,"—is undoubtedly too exclusive; for the veins certainly take up many substances, both from the intestinal tube and the external surface; and it may be questioned whether the lacteals are concerned in anything else than nutritive absorption, and whether the lymphatics have not for their function to absorb those products of the decomposition of the tissues which are to be re-introduced into the nutritive apparatus, rather than those which are destined for secretion, and which are pretty certainly returned by the veins. A long note is appended to these preliminary observations, claiming for Dr. W. Hunter the discovery of the true origin, course, and functions of the lymphatic system, which had been attributed by some of his detractors to Noquez, whose ideas on the subject were evidently very imperfect.

The series of the Circulating System commences with those which exhibit the circulation in vessels without a heart, evidently the true way of showing that the movement of blood through capillaries does not depend upon *vis a tergo* alone. In this division is the preparation from which is taken Mr. W. Bell's very beautiful drawing of the vascular system of the amphinome, one of the marine annelida; although no description of this drawing was left by Hunter, yet it is evident that he must have thoroughly understood the course of the circulation, so beautifully is the whole system of vessels displayed. The portion of the series illustrating the various types of the heart's structure is most beautiful and complete; many rare preparations have been added within a recent period; and the descriptions of all the more important ones are sufficiently complete to serve as valuable data to the student of comparative anatomy. This is especially the case with regard to the hearts of reptiles, which exhibit so interesting a transition from the single to the duplex apparatus of circulation. The anatomy of the siren, in which the respiratory apparatus is double during the whole of life, had received from Hunter a large share of attention; and several preparations of the heart and large vessels are contained in his museum. By Hunter it was believed that the heart in this group possesses only one auricle; and in this error he has been followed by subsequent anatomists, not excepting Cuvier. It has been corrected, however, by Mr. Owen, who has proved that the pulmonary and systemic veins open into distinct auricles. The origin of the mistake appears to have been the fact, that the left or pulmonary auricle is very small; and that the pulmonary veins in reaching it unite into a common trunk, which seems to pass through the great sinus of the veins of the body, but really adheres to the parietes of that sinus by its posterior surface. This remarkable structure led Hunter to suppose that the sinus was part of the pericardium; and that the venæ cavæ opened into it.

In his general remarks upon the Respiratory System, Hunter embodies all those general ideas which were afterwards put forth by Cuvier in his introduction to the Règne Animal, and of which the latter has received the full credit. They display so completely the extent of Hunter's inquiries, and his power of philosophic generalization, that we shall quote them almost in full. The truths they contain are at present familiar to every tyro in physiology; but this was far from being the case fifty years ago:

"Every part of an animal so exposed to the air as for the blood to be affected by it in such a manner as to support life, may be called Lungs, or Respiratory Organ; but what is commonly understood as such is an apparatus formed for that purpose, as a distinct part of the animal. But I conceive it very probable that there are animals so simple in their construction, as not to require a peculiar structure for this purpose. I even know there are many so constructed, where an apparatus of this kind could not be applied, such an apparatus not according with the other parts. Yet I do conceive that in such the application of air is as necessary as in those where an apparatus is found; but where there is a distinct respiratory apparatus, there must be other corresponding apparatuses. Where there is such an apparatus, we find it admits of forms fitted for the different modes of respiration; yet all are included in the terms *branchiæ*

or gills, and *pulmones* or lungs. But there should be a generic term, admitting of divisions into species, so as to be characteristic of the orders of animals to which they belong. Without a collecting and a motion of the nutritive juices, I can conceive there can be no respiratory organ; for I find that the different circulations in the different orders of animals, as far as I know, are so connected with different kinds of lungs, as for either system not to be intelligible alone. In animals, where there is no circulation, there can be no lungs; for lungs are an apparatus for the air and blood to meet, and can only accord with motion of blood in vessels. But where there is no circulation, yet we must suppose, from analogy, that the air affects the juices that are to carry a continuance of life and support to all parts of the body. In the most simple animals, and such as breathe water, the whole apparatus is to have a considerable quantity of very vascular surface brought in contact with the medium in which the animal lives. In the air-breathing orders above fish, there is a simple bag, very vascular, for the reception of air, and this is divided and subdivided as we proceed towards the more perfect animals, till at last the cells are infinitely small. The lungs may be considered, respecting their blood-vessels or circulation, as similar to a gland; for the blood sent to them is not for their own proper use entirely, and indeed only a very small portion of it is for their own use, the larger portion being intended as a secretion from them, as also to receive." (Vol. ii. pp. 66-8.)

The following descriptions we select as good examples of this series, and as probably new to most of our readers:

"1029. The anterior part of a lamprey, showing the seven branchial apertures of both sides, and the corresponding gills of the left side partially exposed. When the lamprey is firmly attached, as is commonly the case, to foreign bodies by means of its suctorial mouth, it is obvious that no water can pass by that aperture from the pharynx to the gills; it is, therefore, alternately received and expelled by the external apertures. If a lamprey, while so attached to the side of a vessel, be held with one series of apertures out of the water, the respiratory currents are seen to enter by the submerged orifices, and, after traversing the corresponding sacs and the pharynx, to pass through the opposite branchiæ, and to be forcibly ejected therefrom by the exposed orifices. The same mode of respiration must take place in the myxine, while its head is buried in the flesh of its prey. The cyclostomous fishes thus present an obvious affinity to the cephalopods, inasmuch as the branchial currents are independent of the actions of the parts concerned in deglutition." (Vol. ii. p. 80.)

"1035. A longitudinal section of the anterior part of the body of a conger eel, showing the branchial cavity and gills, which are minutely injected. The water which is admitted by the mouth passes through five oblique apertures into the branchial cavity, and is forcibly driven by the simultaneous action of the branchial arches and operculum through the interspaces of the gills, and escapes by a single outlet, as in all osseous fishes. This outlet in the conger and other eels is a small vertical fissure, situated at some distance behind the gills; the branchial cavity is therefore proportionately elongated, and the escape of fluid from it is consequently impeded. As the branchial laminæ are thus kept apart and supported by the contained fluid, which insinuates itself everywhere between them, the circulation goes on in them uninterruptedly when the fish is out of the water; and as fresh air is probably absorbed from the surrounding atmosphere, as that which was originally mixed with the water becomes deteriorated by the respiratory process, the fish is enabled by this modification of the branchial apparatus to remain out of its natural element for a considerable length of time." (p. 82.)

The series of preparations of the urinary apparatus is peculiarly extensive and complete. Many of the most interesting of these were made by Hunter at a very early period, being noticed in his first manuscript catalogue. The introductory observations exhibit his full acquaintance with this important branch of comparative anatomy, and his account of the very first preparation shows his remarkable perception of the real analogies of doubtful organs:

"1176. The soft parts of a snail, injected and prepared to show the gland in the respiratory cavity which surrounds the pericardium; the duct may be observed to run along the convex side of the rectum. The following is the description of this preparation in the original manuscript catalogue: 'A snail; shell taken off; air-bag opened; membrane exposed, covering part of viscera and genitals, some coils of which are seen through it; on the right is the last gut, as if a continuation of the spiral turns; and in the semicircular direction is the duct of that gland, running in the doubling of the air-bag. This, I believe, is kidney; its mucus is like that of birds; its opening is near the anus, and accompanies the rectum.' The researches of Professor Jacobson have shown that the secretion of this gland contains urea." (Vol. ii. p. 115.)

The second volume contains seventeen plates, of which the first includes the beautiful drawing of the vessels of the amphinome already referred to, with other subjects illustrative of the first stages in the development of the vascular system. The next four represent the circulating apparatus in the lobster, which Hunter had very carefully examined, and which he described much more accurately than Messrs. Andouin and Milne Edwards have since done. From Hunter's figures and descriptions it appears that both the bloods from the general system, and that from the respiratory organs, are received into a large sinus or auricle surrounding the heart, into the cavity of which they pass by several orifices, and from which they are again propelled, so mixed, both to the general system and to the gills. The French naturalists asserted that the heart of the crustaceans is a true systemic one, receiving only the aerated or pure arterial blood from the branchial veins, which terminate by two orifices in the sides of the ventricle, and these they assert to be the only venous orifices of the heart. The accuracy of Hunter's description, however, is corroborated by the subsequent dissections of Lund and Straus-Durckheim; and it is demonstrated by a preparation made by Mr. Owen, (No. 898 A,) in which black bristles are passed transversely through the four venous orifices that give passage to the blood of the general system from the great sinus surrounding the ventricle, these orifices (represented by the French anatomists as mere depressions) being respectively guarded by two semilunar valves. The succeeding plates depict the circulating and respiratory apparatus of the solen, pearly nautilus (by Mr. Owen); cuttle-fish, menopoma (one of the perennibranchiate batrachia, aptly denominated *pneumobranchiata* by Hunter) whose position between the siren and the ordinary batrachia is exactly assigned, common fowl, ostrich, crocodile, porpoise, &c. In the description of the fauces of the crocodile, the mechanism is distinctly pointed out, by which the communication between the mouth and fauces is closed, and the apertures of the larynx and pharynx defended both against the insects and other parasitic animals, which gain admittance to the mouth from its being unprotected by lips, and also against the entry of water during the period when the crocodile holds submerged a living and struggling prey. The credit of the discovery of this peculiarity has usually been assigned to the French savans who accompanied Napoleon in his Egyptian expedition.

The third volume contains the Nervous System and Organs of Sense, and the Tegumentary System; thus completing the first division, that of "organs in plants or animals for the special purposes of the individual." There is also a series of "peculiarities," embracing many interesting preparations which could not be well disposed elsewhere. It is justly re marked by the editor, that

"There is perhaps no department of anatomy in which greater advances have been made since the time of Hunter, than in the nervous system; both as regards the knowledge of the details of the various modifications which it presents in the animal kingdom, and with respect to the determination of its functions. It could hardly be expected, therefore, that his observations on this system, or any of its parts, would be characterized by the same depth and completeness as are manifested in his writings and preparations relative to the structures in which he was more immediately interested, viz. those relating to the vital functions. Nevertheless, the following pages will show that he had at least obtained a glimpse of all the leading modifications of the sensitive apparatus, and had accurately traced the component parts of the brain through their singular metamorphoses in the highest classes of animals without losing sight of their true analogies; of which, indeed, had the present manuscript been published in the lifetime of the author, it would have contained the earliest enunciation." (Vol. iii. p. 1.)

We cannot stop to advert to all the passages which bear out this statement: but the following strikes us as among the most interesting. In Hunter's preliminary observations, there is a classification of animals founded upon the structure of their nervous system; and the first division embraces "the first class of animals that have organs of sense, and consequently have brains." The preparations which in the original MS. catalogue illustrate the condition of the nervous system characteristic of this "class" are derived exclusively from the molluscous sub-kingdom of Cuvier; whence it may be inferred that Hunter had a perception of that great natural division of the animal kingdom, more especially as his other "classes" are extremely well defined, and embrace all other animals, save the radiata, of the existence of a nervous system in which, Hunter does not seem to have been aware. The following is Hunter's description of the nervous centres in the gasteropod mollusca, (from which alone his preparations are derived;) its correctness will be evident to any one familiar with thesubject.

"The brain in this class of animals is scarcely similar in any respect to that of the most perfect animals with which we are in general more acquainted. It consists of a pulpy substance, somewhat transparent, which is easily squeezed out when the brain is cut into. It appears in some, and perhaps in all the lower classes that have brains, in the shape of a ring, from the circumference of which arise the nerves as radii from a centre. Through this ring (in such) passes the œsophagus. I am apt to believe, however, that this ring is not wholly brain, but a union of two large lateral nerves, which unite under the œsophagus. This at least appears to be the case with the next class. It is not inclosed in hard parts, and is not defended from pressure or injuries more than any other internal part." (Vol. iii. p. 5.)

When speaking of the brain of fishes, Hunter remarks that the void space within the skull is filled with a cellular membrane, analogous to the tunica arachnoides; the accuracy of which account is now universally acknowledged. The purpose of this arrangement is evidently to give to the head the large size which it requires for dividing the water, and for the attachment of muscles, without incurring an undue accumulation of ponderous matter about the brain. After describing the general characters of the brain of reptiles, he remarks: "Although the crocodile is classed with the amphibia, (of Linnæus,) and really comes nearer to that class than to any other that I know of, it has not all the same character, as has been observed. It comes nearer the bird than any of the other amphibia, and therefore is a degree higher. The brain, although it has the same parts, yet it has them closer connected, and the skull is more in contact with it." The justice of this observation is confirmed by the fact, that most modern naturalists have separated the crocodile from the ordinary saurians, and assigned to it a higher place.

Several highly interesting preparations have been added to this series by Mr. Owen and others. Amongst these we shall notice two, (Nos. 1302 A, and 1303 B,) which were specially referred to in Mr. Owen's recent Lectures on the Nervous System, as having an important bearing on the question of the respective functions of the ganglionic and non-ganglionic tracts in the articulata. It is well known that, in the estimation of Dr. Grant and Mr. Newport, the ganglionic tract is sensory, the non-ganglionic motor; whilst Dr. Carpenter has laboured to prove—by comparison of the nervous system of the articulata with that of mollusca, as well as by an examination of the phenomena presented by the former animals after a division of the ganglionic column,—that the ganglionic portion is the centre of reflex action, the non-ganglionic, the channel of sensori-volitional action of which the cerebral ganglia are the real centres. The following are Mr. Owen's remarks on this subject, as reported in the Medical Times, (June 25, 1842:)

"We have before us two opposite conditions of a large and important part of the trunk of two nearly allied and similarly-organized crustacea. In one, the lobster, the post-abdomen is encased in a series of calcareous rings, forming a hard and insensible chain armour. But in the same degree as sensibility is lost the muscular power is increased; a great proportion of the contractile fibre is concentrated in the tail of the lobster, which forms its most powerful and almost exclusive organ of swimming. In the pagurus (hermit-crab) on the other hand, the muscular system is almost abrogated in the long post-abdomen, for this in fact takes no share in the locomotive functions of the body. It is occupied by part of the alimentary canal, and by glandular organs; the external integument has no part of its sensibility destroyed by the interposition of calcareous particles, but retains the necessary faculty of appreciating the smooth and unirritating condition of the interior surface of the deserted shell which it chooses for its abode; nay more, minute acetabula are developed in groups upon this sensitive integument; delicate ciliated processes are also attached to it, to which the eggs adhere in clusters, during their incubation in the female. The muscular system is reduced to a few minute fasciculi of fibres, regulating the action of the terminal claspers. If, as has been conjectured, the ganglionic enlargements of the abdominal cords monopolize the sensorial functions, and the non-ganglionic tracts the motor powers, we ought to have found no ganglia in the tail which is constructed for motion exclusively; whilst in the tail which is almost as exclusively sensitive, the ganglia ought to have been large and numerous. The contrary, however, is the fact. Six well-developed ganglia distribute nerves to the muscular fibres of the lobster's tail; non-ganglionic columns supply the sensitive tail of the hermit crab, the only ganglion in which is the small terminal one, that seems to have been called into existence solely to regulate the actions of the muscles of the organ of adhesion. .... Admitting from analogy that the supra-œsophageal ganglion is that in which true sensation and volition reside, then those nervous filaments, which are exclusively connected therewith, and some of which would seem to extend the whole length of the animal along the dorsal aspect of the ganglionic columns, would form with their ganglionic centre the true sensori-volitional system; whilst any other ganglia superadded to the abdominal columns, with the nervous filaments terminating in or originating from them, would constitute the system for the automatic reception and reflection of stimuli. In these views I coincide with the ingenious physiologist Dr. Carpenter, and shall feel happy if their accuracy and soundness have received any additional proof from the facts of comparative anatomy, now for the first time, I believe, brought to bear upon this interesting problem."

Of the numerous interesting preparations contained in the series on the Organs of Sense, our space only permits us briefly to notice the one which remains as a record of Hunter's discovery of the distribution of branches of the fifth pair to the olfactory surfaces. This preparation (No. 1550) was made in 1754, and is probably the oldest in the museum. It is described and figured in the "Animal Economy," where its history is given; and the same paper contains many interesting suggestions as to the objects of the complete distribution of the nerves, some of which stimulated Sir Charles Bell (we have his own authority for stating) to his successful researches. The following are the ingenious remarks of Hunter, immediately connected with this subject:

"In this dissection I found several nerves, principally from the fifth pair, going to and lost upon the membrane of the nose; but suppose that those have nothing to do with the sense of smelling,—it being more than probable that what may be called organs of sense have particular nerves, whose mode of action is different from that of the nerves producing common sensation, and also different from one another,—and that the nerves on which the peculiar functions of each of the organs of sense depend are not supplied from different parts of the brain. The organ of sight has its peculiar nerve; so has that of hearing, and probably that of smelling likewise; and on the same principle we may suppose the organ of taste to have a peculiar nerve. Although these organs of sense may likewise have nerves from different parts of the brain, yet it is most probable such nerves are only for the common sensations of the part, and other purposes answered by nerves. Thus we find nerves from different origins going to the parts composing the organ of sight, which are not at all concerned in the immediate act of vision; it is also probable, although not so demonstrable, that the parts composing the ear have nerves belonging to them simply as a part of the body, and not as the organ of a particular sense; and if we carry this analogy to the nose, we shall find a nerve which we may call the peculiar nerve of that sense, and the other nerves of this part, derived from other origins, only conveying common sensation, and we may suppose only intended for the common actions of the part. This mode of reasoning is equally applicable to the organ of taste; and if the opinion of peculiar nerves going to particular organs of sense be well founded, then the reason is evident why the nose, as a part of our body, should have nerves in common with other parts, besides its peculiar nerves; and as the membrane of the nose is of considerable extent, and has a great deal of common sensation, we may suppose the nerves sent to this part for that purpose, will not be few in number. It is upon this principle the fifth pair of nerves may be supposed to supply the eye and nose in common with other parts." (Vol. iii. p. 96.)

How much sounder and more philosophical is such reasoning, than that by which Magendie erected upon his ill-devised and ill-observed experiments the absurd dogma that the fifth pair is the real nerve of smell.

We believe that to Hunter is really due the first discovery of the organ of hearing in fishes, which he states himself to have demonstrated some time before he quitted his anatomical pursuits on going with the army to Belleisle, in 1760. It appears that scientific piracy was not uncommon even in those unenlightened days. "As in this age of investigation," says Hunter, "a hint that such an organ existed would be sufficient to excite a spirit of conjecture or inquiry, I was aware that there would not be wanting some men who, whether they only imagined the fact might be true, or really found it to be so, would be very ready to assume all the merit of the discovery to themselves." The following observation fully demonstrates his philosophical perception of the great law of unity of plan, to which all the various modifications of organs are referrible; although it is nowhere else perhaps so distinctly enunciated, yet in many other places there is obvious indication that Hunter was equally cognizant of its application to all departments of comparative anatomy: "I am still inclined to consider whatever is uncommon in the structure of this organ in fishes, as only a link in the chain of varieties displayed in its formation in different animals, descending from the most perfect to the most imperfect, in a regular progression."

We cannot stop to notice any of the interesting preparations in the series of "tegumentary organs" or of "peculiarities;" and we can only add with respect to this third volume, that it contains eighteen plates illustrating some of the most interesting subjects in it.

The two remaining volumes are appropriated to the second division; and include, therefore, a description of the preparations illustrating the Reproductive Function. From the large number of these preparations contained in the museum, it is evident that Hunter had given special attention to the subject; and his preliminary observations to the first subdivision, which contains the organs of generation, afford a masterly though brief exposition of the different modes in which this function is performed. We have already so nearly approached our limits, however, that we must pass by the fourth volume with this brief notice, merely stating that the descriptions it contains are not less interesting than the preceding, nor the preparations themselves less worthy of study. In fact they furnish a suite in itself almost complete enough to serve as the foundation for a full account of the comparative anatomy and physiology of the generative system. We must not omit to add that the volume contains eighteen plates, one of which gives another beautiful representation of the holothuria; whilst several others depict, not less elaborately, the female generative organs of fishes, the many curious types of which afford such interesting objects of study to the philosophic anatomist.

The fifth and last volume contains the second subdivision of the series on Reproduction; which relates to the products of the generative organs, and to the accessory structures and secretions of the parent, which are essential or auxiliary to the development and growth of the offspring prior to its acquisition of independent powers of existence. The manuscript catalogue of this part contained none of those general observations, designed to exhibit the scope and results of the author's researches, which the preceding divisions possessed; but the deficiency has been admirably supplied by Mr. Owen, who has given in an introduction a very complete summary of the contents of the volume. The first series of preparations relates to the structure of fruits; and contains a series of dissections which the botanist may consult with advantage, so numerous are the forms presented, and so beautifully are the most important parts displayed. The preparations illustrating the development of the radiated and molluscous classes are not so numerous as could be wished; there is, however, a very beautiful series illustrating the progressive stages of formation of the ovum of the cephalopods, the development of the embryo, and the peculiar place of attachment of the pedicle of the vitelline sac. The collection of insect dissections, however, is perhaps a more striking illustration of Hunter's unwearied perseverance, and of his determination thoroughly to explore the mysteries of the subject, than any other in this series. It includes upwards of forty preparations displaying the development of the ovum and metamorphoses of the lepidopterous insects; the external and internal peculiarities of their larva, pupa, and imago, being illustrated principally by dissections of the silk-moth. An analogous series of forty-five preparations illustrates the whole history of the generative function in the bee tribe; a part of these are described in Hunter's celebrated memoir on the hive-bee (Phil. Trans. 1792); but the corresponding series, displaying the economy of the humble-bee, is similarly illustrated in a manuscript account of experiments and observations, published for the first time in the volume before us. This beautiful memoir affords an excellent example of the mode in which the habits and general economy of insects ought to be studied. The development and metamorphoses of insects belonging to the other orders are scarcely less fully displayed, as may be inferred from the fact that the number of preparations illustrating the function of reproduction in the entire class is not less than 480.

The numerous preparations of the gravid ovaries, ova, and young of fishes, and especially those relating to the long unknown and much disputed subject of the propagation of the eel and lamprey, receive the same additional interest and value from the manuscript relating to them, as is conferred by Hunter's written record of his lucid and philosophical views upon the other series of preparations that are similarly illustrated. His original observations, included in the present volume, advocate the oviparity of the eel and lamprey from the anatomical conditions and analogies of the female organs; and the accuracy of the views of Hunter has been subsequently confirmed by the more direct observations on the generation of the eel, made by Davy, and by those experienced icthyologists, Couch and Yarrell. The curious peculiarities in the generation of the cartilaginous and lophobranchiate fishes are not less completely illustrated. Nor are the circumstances attending the development of the embryo in the class of reptiles less fully displayed. Most of the external and internal changes accompanying the metamorphosis of the newt and frog are displayed in elaborate dissections and entire specimens of the larva at each gradation of growth; and with Hunter's accurate knowledge of the anatomy of the siren, amphiuma, and menophoma, the analogy between the permanent states of the latter, and the phases of development in the former could not have escaped him.

One of the most interesting series of preparations in the whole museum, however, is that which illustrates the development of the bird's egg. It contains fifty specimens; and many of these were delineated by W. Bell, whose drawings have been engraved for the present volume. Of these drawings we cannot speak too highly. They combine every quality that could be desired in the representation of such subjects, and are immeasurably superior to any other delineations we have seen. Their truthfulness must be evident at a glance to any who are acquainted with the objects they represent; and they will bear the closest examination as to details. When it is considered that these drawings were executed between fifty and sixty years ago, it does not speak very highly for the liberality or judgment of the Council of the College, that they have been so long withheld from the world; since their publication would have been not merely an important boon to the student of embryology, but would have at once established Hunter's claim to the character of a great discoverer. Three of the figures were engraved, but in a very inferior style, for the treatise on the Blood; but the account of them given by the posthumous editor is very imperfect and erroneous. Among the points which they clearly demonstrate is Hunter's knowledge of the existence of the so-called "serous lamina" of the germinal membrane, and of the mode in which the amnion is formed from this; a discovery usually attributed to Pander, whose thesis on the Development of the Egg was published in 1817. The production of the red corpuscles subsequently to the commencement of the circulation of liquor sanguinis is another of Hunter's important observations, since claimed by others, although clearly stated in the treatise on the Blood. "I well remember," says Mr. Owen, "the feelings of surprise with which I listened, while at Paris in 1832, to a memoir read before the Academy of Sciences by MM. Delpech and Coste, the object of which was the announcement of the same fact as a novel and important discovery. The statement of the French observers was received with all the consideration which its importance justly merited, without its being suspected that our great physiologist had half a century before embraced it, with all its legitimate deductions, in the extended circle of his investigations." One of the most remarkable "anticipations" in the paper on the Development of the Egg, is contained in the following passage: "If we were capable of following the progress of increase of the number of parts of the most perfect animal, as they first formed in succession, from the very first to its state of full perfection, we should probably be able to compare it with some one of the incomplete animals themselves of every order of animals in the creation, being at no stage different from some of the inferior orders; or, in other words, if we were to take a series of animals, from the more imperfect to the perfect, we should probably find an imperfect animal, corresponding with some stage of the most perfect." Again, he says in another place; "We may also observe that the first rudiments of every animal are extremely soft; and even the rudiments of the more perfect are similar to the full-grown imperfect, and as they advance in growth, they become firmer and firmer in texture." It is well known that the deficiencies of Hunter's early education placed him under great disadvantages in regard to the expression of his ideas, particularly when these were of a general and abstract kind; and this we conceive to have been peculiarly the case with respect to the important generalization in question, which, though now universally recognized, must have seemed to Hunter and his contemporaries a daring novelty.

With these observations we must now close our notice of this truly splendid work, which ought not only to redeem our country from the discredit of being the last to take up the study of anatomy and physiology in a philosophical spirit, but to give satisfactory evidence that the true progress of these sciences is to be dated from the time of Hunter. There is evidence that not only Poli, but Camper, Scarpa, and

Blumenbach, profited by those occasional prelections, in which he explained to some chosen minds which could respond to the conceptions of his own, his great scheme, embracing the demonstration of all the leading modifications of every organ of the animal body, and of the different stages which each organ undergoes in its development, to fulfil the functions it is required to perform in the highest organisms. In estimating, therefore, the share which Hunter had in advancing comparative anatomy and physiology, we must not overlook the influence which these expositions must have had on the subsequent labours of such pupils.

But it is in his Museum that we are to seek the fullest and widest evidence of the advance made by Hunter in the exploration of the *penetralia* of the science of life. It is here that we best discern his unwearied industry in the collection of facts, his sagacity in the grouping together of analogous phenomena, his philosophic grasp of sound inductions, and his intuitive perception of truths which he had not the means of fully demonstrating. "Had the means and time been granted to Hunter to have made public the results of all his labours, or had his manuscripts enunciating, or indicating, so many general principles, been fairly appreciated and given to the world, our teachers of anatomy would not now, after the lapse of half a century, have but begun to explain to their students those beautiful laws of animal development, for the knowledge of which they are indebted to the labours of the professors in the noble schools of physiology in continental Europe, where the spirit of Hunterian inquiries seems to have long so exclusively resided. But the period which has elapsed before those general laws began to be appreciated in the country where they were first detected affords, perhaps, one of the strongest indications of the great advance which Hunter had made in physiological science."*

We have a word to say, in conclusion, to the Council of the College of Surgeons, under whose direction Mr. Owen's labours have been devoted for many years to this splendid work: although its price has been fixed (as we understand) at so low a rate, as barely to meet the cost of print and paper, yet its cost is great enough to put it out of the reach of the class who should most profit by it,—students and junior practitioners. To many of those who would receive from it the highest delight and the greatest benefit, we fear it will remain a sealed book. Now when it is considered that the property of the college is virtually the property of all its members, and not of the council alone, that its funds are derived from the contributions of its members, and that the whole profession, therefore, has already defrayed the expense of the work, we think it would be an act of bare justice to them that they should have the opportunity of possessing themselves of it, at a price little more than nominal. Even if the council have cogent reasons for denying this, they might still do great service by presenting a few copies to the libraries of medical or scientific institutions; and thus in some degree compensating the inhabitants of the provinces for their want of power to consult the museum itself. Such libraries are seldom in a flourishing state, as regards their funds; and the purchase of a work of this kind usually involves the necessity of foregoing others of perhaps more practical importance. We trust that our hint will be taken in good part, whether or not it is attended with the desired effect.

* Prof. Owen, in Introduction to vol. iv. of Palmer's edition of Hunter's works.

